# Thyme, Oregano, and Garlic Essential Oils and Their Main Active Compounds Influence *Eimeria tenella* Intracellular Development

**DOI:** 10.3390/ani14010077

**Published:** 2023-12-25

**Authors:** Martina Felici, Benedetta Tugnoli, Callum De Hoest-Thompson, Andrea Piva, Ester Grilli, Virginia Marugan-Hernandez

**Affiliations:** 1 DIMEVET, Dipartimento di Scienze Mediche Veterinarie, Università di Bologna, Via Tolara di Sopra 50, Ozzano dell’Emilia, 40064 Bologna, Italy; andrea.piva@unibo.it (A.P.); ester.grilli@unibo.it (E.G.); 2 Vetagro S.p.A., Via Porro 2, 42124 Reggio Emilia, Italy; benedetta.tugnoli@vetagro.com; 3 Department of Pathobiology and Population Sciences, Royal Veterinary College, University of London, Hatfield AL9 7TA, UK; cdehoestthomp22@rvc.ac.uk (C.D.H.-T.); vhernandez@rvc.ac.uk (V.M.-H.); 4 Vetagro Inc., 17 East Monroe Street Suite #179, Chicago, IL 60603, USA

**Keywords:** avian coccidiosis, *Eimeria tenella*, in vitro, schizogony, botanicals, anticoccidials

## Abstract

**Simple Summary:**

Anticoccidial agents, crucial for controlling parasitic infections in poultry, should ideally interfere with multiple stages of the parasite’s lifecycle. This study aimed to evaluate the influence of anticoccidial drugs, natural essential oils, and their pure bioactive compounds on the first round of schizogony of *Eimeria tenella* in vitro. The results revealed that both essential oils and conventional anticoccidial compounds were equally adept at preventing schizont formation. However, the pure bioactive compounds displayed only a slightly reduced level of development, indicating a potential decrease in pathogenicity. This investigation sheds light on the capacity of natural substances to disrupt the intracellular development of the *E. tenella* parasite, providing valuable insights into their mechanisms of action and their potential for safer alternatives in anticoccidial development.

**Abstract:**

Coccidiosis poses a significant challenge in poultry production and is typically managed with ionophores and chemical anticoccidials. However, the emergence of drug resistance and limitations on their use have encouraged the exploration of alternative solutions, including botanical compounds and improvements in in vitro screening methods. Prior research focused only on the impact of these alternatives on *Eimeria* invasion, with intracellular development in cell cultures receiving limited attention. This study assessed the impact of thyme (*Thymus vulgaris*), oregano (*Origanum vulgare*), and garlic (*Allium sativum*) essential oils, as well as their bioactive compounds, on the initial phase of schizogony in Madin–Darby bovine kidney cells, comparing their effectiveness to two commercially used anticoccidial drugs. Using image analysis and quantitative PCR, the study confirmed the efficacy of commercial anticoccidials in reducing invasion and schizont formation, and it found that essential oils were equally effective. Notably, thymol and carvacrol exhibited mild inhibition of intracellular replication of the parasite but significantly reduced schizont numbers, implying a potential reduction in pathogenicity. In conclusion, this research highlights the promise of essential oils and their bioactive components as viable alternatives to traditional anticoccidial drugs for mitigating coccidiosis in poultry, particularly by disrupting the intracellular development of the parasites.

## 1. Introduction

Avian coccidiosis is one of the most concerning parasitic diseases as it poses a significant threat to the health of birds and the poultry industry, which is estimated to be up to USD 13 billion per year [1]. *Eimeria* spp. are recognized as the causative agent of the disease, and they are protozoan parasites that replicate inside the host’s enterocytes, causing inflammation, ulceration, and bleeding. This results in poor nutrient absorption and growth. High mortality rates are also common in severe cases, while, in sub-clinical cases, an infection with *Eimeria* spp. can predispose to secondary opportunistic pathogen infections, such as *Clostridium perfringens*, *Campylobacter jejuni*, and *Salmonella infections* [2,3,4]. 

The treatment and prevention of coccidiosis relies on the use of ionophores and chemical anticoccidials; however, spreading of resistance due to their extensive use has raised concerns in the poultry industry [5]. Significant research efforts have been directed towards exploring alternatives to anticoccidials. Particularly, attention has been focused on botanical compounds and natural remedies, which allegedly exhibit the ability to disrupt multiple stages of parasite development, offering promising avenues for combating this parasitic disease [6]. 

Among the various compounds that have been tested, essential oils (EOs) derived from Lamiaceae and Amaryllidaceae (i.e., *Oregano* and *Allium* spp.) have shown positive outcomes in alleviating the clinical signs of coccidiosis in vivo and in reducing oocyst shedding after infection. In addition, in some cases, an improvement in the *Eimeria*-induced lesions has been assessed [7,8,9]. The positive effects of these substances are believed to be due to the bioactive molecules they contain. Thymol and carvacrol are two of the main monoterpene phenols of Lamiaceae plants, like thyme and oregano. They have well-documented antibacterial and immunomodulatory proprieties, which make them good candidates to treat enteric infections [10,11]. Also, recent studies have claimed them as anti-protozoal agents against *Lehismania* spp. and *Cryptosporidium parvum* [12,13]. *Allium* spp. contain a wide pool of biologically active organosulfur compounds, including allin, ajoene, allicin, diallyl sulphide, and S-allylcysteine, showcasing antibacterial, anti-inflammatory, antiseptic, antiparasitic, and immunomodulatory properties [14,15].

However, due to their high variability, these botanical mixtures are far from being adequately characterized, especially on their anticoccidial proprieties and modes of action. To do so, an improvement of the available screening methodologies for novel anticoccidial candidates is needed.

The challenges associated with obtaining reliable and standardized in vitro models have led to a predominant reliance on in vivo research methods for studying these alternatives to ionophores and chemical anticoccidials. However, conducting research in vivo presents certain limitations, including the intricate and variable nature of these models and ethical concerns associated with animal experimentation, as well as the high cost and restricted availability of necessary resources [16].

Among the various species causing chicken coccidiosis, *E. tenella* holds particular significance due to its high pathogenicity. For this reason, it has been the most studied avian *Eimeria* species. Consequently, it serves as a valuable model for studying and conducting research on these parasites in vitro [16]. In addition, unlike other species, *E. tenella* is able to undergo the first round of schizogony in immortalized mammalian cell lines, like Madin–Darby bovine kidney (MDBK) cells [17]. This property has been used in previous studies to establish a robust in vitro assay for evaluating the first round of schizogony [18]. Moreover, researchers utilized a transgenic fluorescent strain of *E. tenella*, which expresses yellow fluorescent protein (YFP), to visually examine the developmental timeline and the impact of certain anticoccidial drugs [18].

In the present study, this methodology has been used to examine the anticoccidial proprieties of three essential oils derived from thyme (*Thymus vulgaris*), oregano (*Oregano vulgaris*), and garlic (*Allium sativum*), and their main bioactive compounds (‘nature-identical compounds’, NIC), on the development of *E. tenella*. Also, the effects of these natural compounds have been compared to two common anticoccidial drugs (AC): salinomycin, an ionophore drug that alters the membrane gradient, and robenidine, a guanidine derivative whose main action is the inhibition of maturation of 1st generation schizonts [19].

## 2. Material and Methods

### 2.1. Parasites and Birds

The strain *E. tenella* dYFP, derived from the Wisconsin strain [20], was propagated in 3-week-old White Leghorn chickens reared under specific pathogen-free conditions [21]. Oocyst harvest, excystation, and sporozoite purification were conducted following the methods described in a previous study [22]. Freshly purified sporozoites (0.1 × 10^6^ per replicate) were used to infect a cell monolayer in a 96-well plate format.

### 2.2. Cell Culture

Madin–Darby bovine kidney cells (MDBK-NBL-1, ECACC-Sigma-Aldrich, Salisbury, UK) were maintained in cell culture flasks in Advanced DMEM (Cat. #11520436—Thermo Fisher Scientific, Waltham, MA, USA) supplemented with 5% fetal bovine serum (Cat. #11573397—Thermo Fisher Scientific, Waltham, MA, USA), 1× penicillin/streptomycin solution (Cat. #11548876—Thermo Fisher Scientific, Waltham, MA, USA), and 1× Glutamax (Cat. #11574466—Thermo Fisher Scientific, Waltham, MA, USA). Four hours prior to invasion, the cells were seeded in 96-well plates (Cat. #10687551—Thermo Fisher Scientific, Waltham, MA, USA) at a concentration of 0.05 × 10^6^ cells/well.

### 2.3. Chemicals and Reagents

Salinomycin from *Streptomyces albus* (Cat. # S4526, Sigma-Aldrich, St. Louis, MO, USA) and robenidine hydrochloride (Cat. # 33979, Sigma-Aldrich, St. Louis, MO, USA) were resuspended, respectively, with ethanol and dimethyl-sulfoxide (Cat. #D2650, Sigma-Aldrich, St. Louis, MO, USA) and used at a final concentration of 1 and 5 ppm, respectively.

All EOs in this study were analyzed at the same concentration (40 ppm), and the NIC were tested at 20 ppm. The choice of a single concentration was based on a previous study [23].

Oregano essential oil (OEO) was provided by Galen-N (Galen-N Ltd., Sofia, Bulgaria). Garlic oil (GAR) was purchased from Lluch Essence (Lluch Essence S.L.U, Barcelona, Spain) and thyme essential oil (TEO) from Grupo Indukern (Grupo Indukern, Madrid, Spain). All the stock solutions were prepared in ethanol and added to cells, with a final concentration of 40 ppm in supplemented Advanced DMEM. In all cases, the final concentration of ethanol was less than 0.5% (*v*/*v*).

Carvacrol (CAR—Cat. #W224511, analytical grade 99%, Sigma-Aldrich, St. Louis, MO, USA), diallyl-disulfide (DDS—Cat. #SMB00378, analytical grade ≥ 98%, Sigma-Aldrich, St. Louis, MO, USA), and thymol (THY—Cat. #T0501, analytical grade ≥ 98.5%, Sigma-Aldrich, St. Louis, MO, USA) were all diluted in ethanol to prepare stock solutions and used at a final concentration of 20 ppm in supplemented Advanced DMEM.

### 2.4. Development Assay

After purification, the sporozoites were added to the cells in the absence or presence of the described treatments. The cells were incubated at 41 °C with 5% CO_2_. After 4 h of incubation, the non-invading sporozoites were removed from the wells, and the medium with the treatments was replenished. After 48 h, the cells were provided with fresh medium along with the respective treatments.

After 24, 48, 72, and 96 h post-infection (hpi), the cells were either fixed in 4% paraformaldehyde or harvested with RTL buffer (Qiagen, Hilden, Germany), as described by the manufacturer’s protocol, for subsequent nucleic acid extraction. Each experiment was repeated at least twice.

### 2.5. Isolation of Nucleic Acids

Genomic DNA (gDNA) was isolated from the samples stored in RTL buffer. The AllPrep DNA/RNA 96 Kit (Cat. #80311—Qiagen, Hilden, Germany) was used for the isolation process, following the manufacturer’s instructions. A vacuum manifold designed for processing 96-well plates (Cat. #9014579—Qiagen, Hilden, Germany) was employed for this purpose.

### 2.6. Real Time Quantitative PCR (qPCR)

To perform qPCR, the CFX96 Touch R Real-Time PCR Detection System (C1000—Bio-Rad, Hercules, CA, USA) was used. The procedures were carried out according to previously described methods, utilizing the DNA-binding dye SsoFastTM EvaGreen Supermix (Cat. #172-5203—Bio-Rad, Hercules, CA, USA) [24].

For parasite quantification, the number of haploid genomes (equivalent to single sporozoites) per well (four wells per sample as technical replicates) was determined. This quantification was achieved using gDNA-specific primers targeting *E. tenella* 5S rDNA (Fw_5S: TCATCACCCAAAGGGATT, Rv_5S: TTCATACTGCGTCTAATGCAC) [20]. A standard curve of sporozoite gDNA, extracted using the same methods, was generated [25]. This standard curve encompassed gDNA equivalent to 10^7^ genomes, followed by serial dilution until 10^2^ genomes.

The inhibition of invasion at 24 hpi was calculated with the following equation developed by Thabet et al. [26]:

$$\%\;of\;inhibition=100\times \left(1-\frac{number\;of\;E.\;tenella\;gene\;copies\;in\;treated\;sample}{number\;of\;E.\;tenella\;gene\;copies\;in\;non-treated\;control}\right)$$

To compare the replication rates after treatments, excluding the effects of invasion, slopes of the replication lines were calculated between 24 and 72 hpi. The slopes were calculated with the formula below, as previously described by Arias-Maroto et al. [25]:

$$m\;\left(slope\right)=\frac{y\;\left(72\;\mathrm{hpi}\right)-y\;\left(24\;\mathrm{hpi}\right)}{48\;\left(time\;lag\right)}$$

### 2.7. Image Analysis

The paraformaldehyde-fixed samples were observed using a fluorescence microscope (Nikon Eclipse Ti2—Nikon, Tokyo, Japan) at 20×, using Plan fluor Nikon lenses at a wavelength of 490 nm. Six random fields were captured and analyzed with a semi-automated procedure relying on a custom script designed on ImageJ. Each picture was converted into an 8-bit image before applying threshold masks separately and converting the picture into a binary image. On the binary images, two possible values (black or white) were given to each pixel, according to the intensity of the fluorescence. The final particle analysis involved identifying the edges of the black and white structures; this allowed the recording of particle numbers and sizes, both on average and individually. These data were collected and processed for statistical analysis. To assess general parasite growth, the average area, including any sort of stage (sporozoites and schizonts), was calculated for every field. Also, for specific cases where a certain level of growth was observed, the schizont area was calculated separately, assuming as schizonts the particles with an area higher than 50 μm^2^. The rate of schizont growth was calculated as the slope of the regression line between 24 and 72 hpi, as indicated by the formula reported in the previous section.

### 2.8. Statistical Analysis

GraphPad Prism 9.4.1 was utilized for statistical analysis. For the qPCR data, the number of genomes was normalized based on the mean value of the challenged control at 24 hpi of each independent experiment. For all data, the descriptive analysis was conducted, and normality was assessed with the Shapiro–Wilk test (*p* > 0.05). Normally distributed data were analyzed using either a one-way ANOVA (for schizont size) or a two-way ANOVA (for repeated measurements). Non-normally distributed data were analyzed using the Kruskal-Wallis test (for schizont number) or mixed-effects analysis (for repeated measurements). To define significant differences among treatments, post hoc multiple comparison analyses were performed: the mean of each treatment was compared with the mean of every other treatment using Tukey’s test for normally distributed data and mixed-effects analysis, while Dunn’s test was adopted for non-normally distributed data. Differences were considered significant when the *p* value was ≤0.05 and were denoted as letters.

## 3. Results

### 3.1. Characterization of E. tenella Intracellular Growth in MDBK Cells

**Figure 1:**
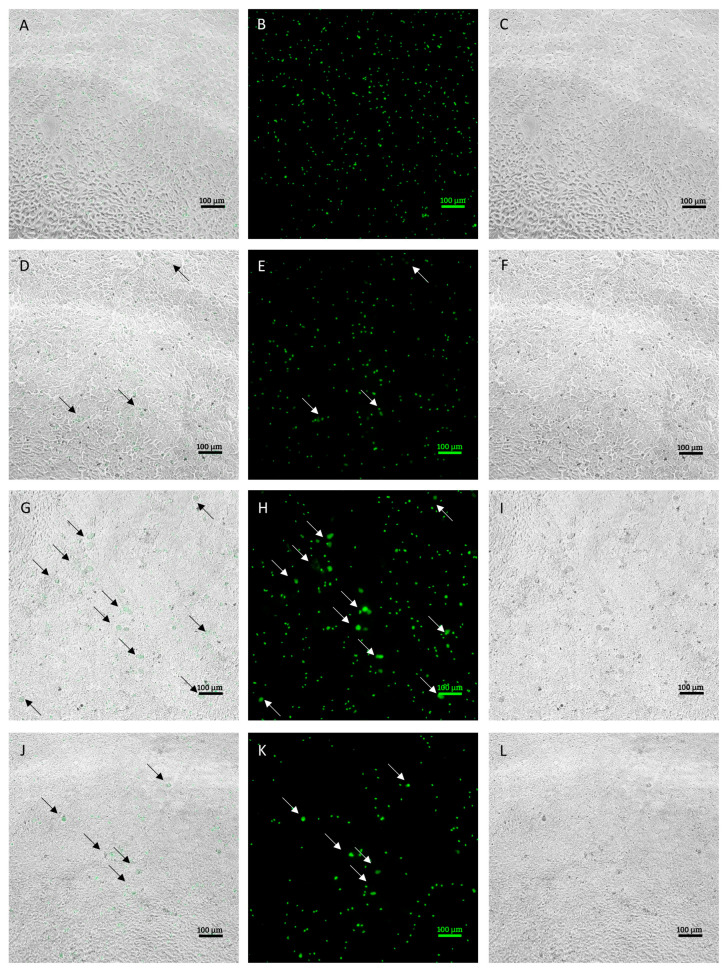
Intracellular development time course (20× objective). Images (**A**–**C**) indicate the intracellular sporozoites at 24 h post infection (hpi) ((**A**) = merge, (**B**) = green fluorescence, (**C**) = brightfield). Images (**D**–**F**) indicate intracellular sporozoites at 48 hpi and early stages of development, indicated by black arrows in merge pictures and white arrows in green fluorescence pictures ((**D**) = merge, (**E**) = green fluorescence, (**F**) = brightfield). Images (**G**–**I**) indicate intracellular parasite at 72 hpi and late stages of development, indicated by white arrows ((**G**) = merge, (**H**) = green fluorescence, (**I**) = brightfield). Images **J**–**L** indicate intracellular parasites at 96 hpi. Residual developmental stages are indicated by white arrows ((**J**) = merge, (**K**) = green fluorescence, (**L**) = brightfield). Size bar ~100 μm.

Infected MDBK cells were examined using a fluorescence microscope to evaluate the morphological characteristics of the different developmental stages obtained in culture on a 96-h time course. Figure 1 shows representative images of the development at each time point.

During the first phases of infection, occurring within 24 hpi, sporozoites that successfully invaded the cells were observed. At 48 hpi, it was already possible to observe some early stages of development, corresponding to early schizonts. Those appeared as round objects, larger in size compared to intracellular sporozoites. At 72 hpi, larger schizonts were clearly observed in the infected monolayer. Compared to early schizonts, which are difficult to distinguish using bright field, late ones appeared more condensed and, in most cases, were clearly discernable in the monolayer. After 96 h, the number and size of schizonts dropped visibly due to schizont breakage.

**Figure 2:**
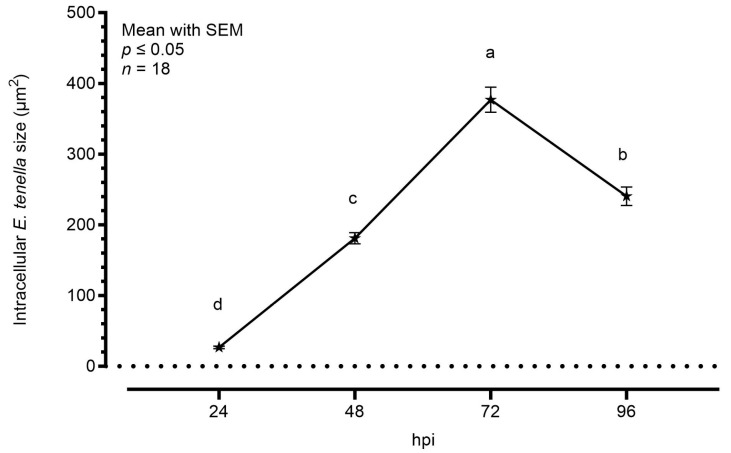
Average size of *E. tenella* intracellular stages measured by semi-automated imaging software. Data are represented as mean ± SEM (*n* = 18). The plotted values are 26.70 ± 1.72 at 24 hpi, 181.00 ± 7.94 at 48 hpi, 377.00 ± 17.91 at 72 hpi, and 240.50 ± 13.01 at 96 hpi. Different letters indicate significant differences among timepoints (*p* ≤ 0.05).

Figure 2 shows the average area increase of intracellular parasites during the time course, considering sporozoites at 24 hpi and only schizonts at later timepoints. Starting from an area of around 30.0 μm^2^, the intracellular sporozoites started their maturation in schizonts. At 48 hpi, the average area was significantly bigger (181.0 μm^2^), but the highest increase was visible at 72 hpi, as the schizonts’ area reached an average of 377.0 μm^2^. At 96 hpi, the area was slightly smaller than the previous timepoint (240.5 μm^2^).

### 3.2. Effects of Natural Compounds on Intracellular Growth

**Figure 3:**
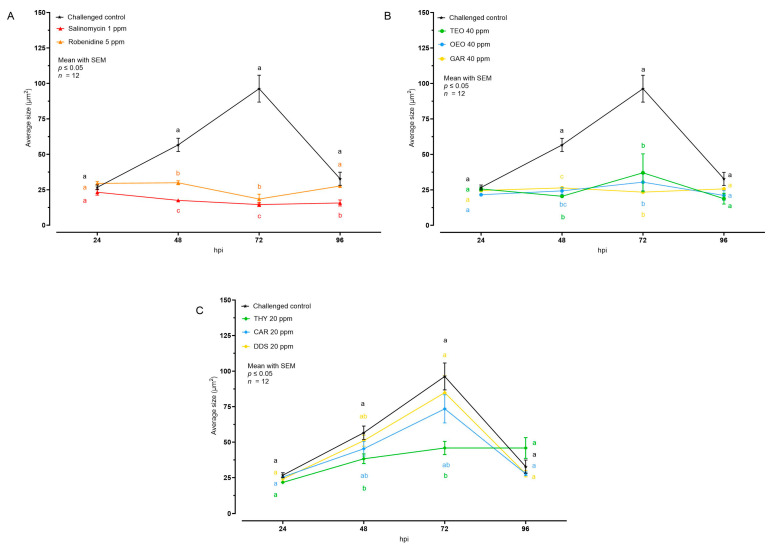
Average size of intracellular *E. tenella* after treatment with anticoccidial drugs (**A**), essential oils (**B**), or nature-identical compounds (**C**). Data are represented as mean with SEM (*n* = 12), and the values are listed in Table 1. Different letters indicate significant differences among groups within the same time point and the same graph (*p* ≤ 0.05). Thyme essential oil (TEO); oregano essential oil (OEO); garlic oil (GAR); thymol (THY); carvacrol CAR); diallyl-disulfide (DDS).

The effect of the different tested compounds on the average size of intracellular *E. tenella* stages is shown in Figure 3 and Table 1. Concerning AC and all the EO, no intracellular development was visible; in the cell monolayer only internalized sporozoites, characterized by an average size of 30 μm^2^, were observed. In the case of NIC, THY 20 ppm allowed a low degree of intracellular development at 48 and 72 hpi, while CAR and DDS did not cause any significant reduction of the average *E. tenella* size compared to the challenged control.

To better understand if the growth velocity was affected by the treatments, the rate of growth was calculated, considering average area values between 24 and 72 hpi (Table 2). In the challenged control group, the intracellular parasites grew by 1.21 μm^2^/h. All AC and EO managed to stop growth, keeping the growth rate close to 0. Among NIC, only THY was significantly lower than the challenged control.

**Figure 4:**
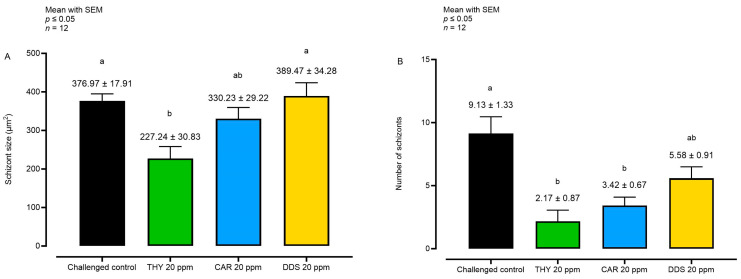
Analysis of schizont size (**A**) and number (**B**) at 72 hpi. Data are represented as mean with SEM (*n* = 12). Different letters indicate significant differences among groups (*p* ≤ 0.05). Thymol (THY); carvacrol CAR); diallyl-disulfide (DDS).

The sizes and numbers of *E. tenella* schizonts were also assessed after treatment in cases where a certain degree of intracellular growth was observed. This was done to examine the impact of these substances specifically on the schizonts at the peak of development, observed at 72 hpi (Figure 4).

Considering the schizont size at 72 hpi, only THY reduced the parameter compared to the challenged control. However, a reduction in the number of sporozoites per field was observable with both THY and CAR, while with DDS, only a non-significant numerical reduction was visible.

### 3.3. Effects of Natural Compounds on Parasite Invasion and Replication

Almost all the compounds significantly reduced the internalization of sporozoites at 24 hpi (Table 3). AC and EO lead to the most significant decrease of invasion, with values above 50% for both groups. Among NIC, only CAR significantly inhibited invasion regarding the control, with a level of 43%.

**Figure 5:**
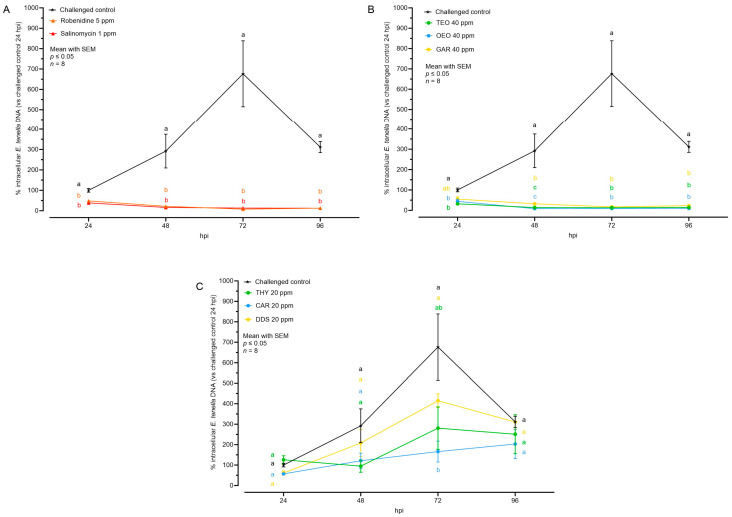
Quantification of intracellular *E. tenella* DNA by qPCR after treatment with anticoccidial drugs (**A**), essential oils (**B**), or nature-identical compounds (**C**). Values are expressed as percentage over the control at 24 hpi. Data are represented as mean with SEM (*n* = 8) and the values are listed in Table 4. Significant differences among groups within the same time point and the same graph are indicated with different letters (*p* ≤ 0.05). Thyme essential oil (TEO); oregano essential oil (OEO); garlic oil (GAR); thymol (THY); carvacrol CAR); diallyl-disulfide (DDS).

Figure 5 shows the quantification of *E. tenella* intracellular genomes as a percentage in relation to the challenged control at 24 hpi. Parasite replication was evident in the challenged control, as the number of genomes nearly tripled every 24 h, peaking at 72 hpi. However, at 96 hpi, the number of intracellular genomes dropped significantly (probably caused by schizont breakage, with release of merozoites into the washed supernatants). 

The ACs and the EOs successfully reduced the number of intracellular parasite genomes starting from the moment of infection (24 hpi). Subsequently, there was no further detected nuclear replication in any of these groups during the subsequent time points (Figure 5). Concerning NIC, a partial inhibition of replication was noticed in the groups THY and CAR. These groups did not exhibit a distinct peak of growth at 72 hpi, suggesting a certain level of efficacy in inhibiting parasite replication. The DDS group did not show any significant difference, but their numbers at the peak (72 hpi) were lower than in the challenged control, potentially influenced by the lower (but not significant) number of invading sporozoites (Figure 5 and Table 4). 

The rate of replication was calculated to investigate the intracellular development velocity of between 24 and 72 hpi (Table 5). In the challenged control group, the replication rate was 6.44 genomes/h. Most of the compounds demonstrated a capacity to reduce this rate. Specifically, AC and EO significantly reduced it, reaching values below 0 (usually caused by non-developing sporozoites observed to leave host cells over time). Meanwhile, THY and CAR managed to decrease the rate to almost half of the challenged control, but not significantly.

## 4. Discussion

Anticoccidials of any nature should be safe for the host, easy to produce and apply, and, preferentially, they should interfere with more than one stage of the parasite’s lifecycle, with a deep and comprehensive characterization of their mode of action [27]. For this purpose, in vitro systems that employ invasion and development assays on cell cultures have been increasingly used, as they allow a closer look at the undergoing processes and higher throughput testing compared to in vivo systems [27]. *Eimeria* spp., in particular *E. tenella*, are able to invade different cell lines and to complete the first round of schizogony in MDBK cells. Moreover, transgenic parasites expressing the YFP fluorescent marker have become a powerful tool for studies on *Eimeria*, especially to study intracellular development in cell cultures, as they allow the direct visualization of intracellular stages (i.e., schizonts) of development [18,27]. 

Previous research has explored the anticoccidial effects of thyme, oregano, and garlic essential oils, as well as their main bioactive molecules, both in vivo and in vitro, focusing primarily on the inhibition of sporozoite invasion [23,28]. Considering that parasite pathogenesis is related to intracellular stages, more extensive evaluation is required to comprehensively assess the impact of these novel compounds. [27]. 

### 4.1. Anticoccidial Drugs

In the present study, the efficacy of salinomycin and robenidine (commercial anticoccidials) has been confirmed, as both were able to decrease the number of sporozoites invading the cell monolayer and also to prevent the formation of schizonts. As previously reported, a pre-treatment with these molecules, before infection, is already enough to cause sporozoites death, thus stopping any further maturation [18]. In a similar study, the impact of 1 h of pre-incubation with salinomycin and robenidine on intracellular development was investigated by qPCR [25]. Pre-treatment with salinomycin 1 ppm lowered the level of invasion by 31%. Additionally, both robenidine and salinomycin pre-treatment managed to decrease the rate of replication between 24 and 44 hpi [25]. In the present study, the treatment with anticoccidials occurred along invasion and continued for the whole time course. This resulted in higher inhibition of invasion at 24 hpi and in no observed replication, suggesting that the anticoccidial power could be time-dependent. 

### 4.2. Essential Oils

Similar to anticoccidials, thyme, oregano, and garlic essential oils were very effective in preventing the formation of schizonts. Inhibition of invasion was observed at 24 hpi with essential oils, suggesting that the action on sporozoites is so detrimental that internalized sporozoites struggle to undergo further development after meeting these compounds. The effect of similar essential oils on invasion has been explored previously by Sidiropoulou and colleagues [9,28]. *E. tenella* sporozoites were pre-treated for 1 h with different concentrations of garlic, oregano, and thyme essential oils from Greece. They recorded effective inhibition of invasion for oregano and garlic essential oils, in particular, at higher concentrations (100 ppm), whereas thyme had more consistent effects at lower concentrations [9,28]. Also, a previous study conducted on primary chicken enterocytes showed that 40 ppm of garlic, oregano, and thyme essential oils effectively reduced invasion at 24 hpi by around 45% [23]. In the first case, differences can be attributed to a different composition of the essential oils. In the second, where the source of the essential oils was the same as that in the current study, the levels of inhibition were slightly lower in the chicken enterocytes compared to MDBK cells. This could be due to differences in the protocols of invasion, but also, since a better predisposition of *E. tenella* to chicken enterocytes has been observed, differences caused by treatments could be dampened [23]. In addition to the cell model used, the effect of these essential oils on invasion and development was clear in the present study. To our knowledge, this is the first study to explore the impact of essential oils and bioactive molecules on *Eimeria* endogenous development in vitro, extending beyond invasion and reaffirming their potential as anticoccidial agents.

### 4.3. Nature-Identical Compounds

The power of essential oils lies in their diverse composition, which encompasses a wide range of bioactive molecules like terpenes, phenols, aldehydes, thiol compounds, and others. Often, these derivatives alone can drive the main effects of the original oil, as they maintain a more uniform composition than essential oils, which depends on specific extraction protocols; therefore, they have become a target of study for their therapeutic effects. In this study, thymol, carvacrol, and diallyl-disulfide, major components of thyme, oregano, and garlic essential oils, respectively, have been investigated to understand if these compounds solely drive the observed effects.

Diallyl-disulfide had no significant impact on development, in contrast to the relative essential oil (garlic), suggesting that the inhibitory effect is likely to be mediated by other molecules within the mixture. Garlic oil is rich in sulfur species that are able to oxidize thiols in protein residues, leading to loss of function. Other studies on sporozoite invasion in vitro evidenced that allicin, found in garlic species too, is a very strong anticoccidial. It shares the main chemical structure with diallyl-disulfide; however, allicin has a thiosulfinate group that makes the molecule very unstable and reactive [29]. A poorer anticoccidial performance of diallyl-disulfide compared to garlic oil was also observed in previous research on *E. tenella* invasion [23], concluding that thiosulfinates could be key effectors to drive the anticoccidial effects.

Thymol and carvacrol slightly reduced the intracellular number of *E. tenella* genomes; in particular, carvacrol reduced by 43% the level of invasion at 24 hpi. A similar effect on inhibition of invasion was observed in a previous study, where a mild level of inhibition was observed on chicken enterocytes, especially for carvacrol [23]. However, in the present study, thymol and carvacrol did not completely stop replication. Nevertheless, they proved to be effective in reducing both the number and size of developed schizonts at 72 hpi, factors associated with decreased pathogenicity, as seen in attenuated *Eimeria* strains [30]. Hence, these treatments may not completely halt development, but they can still result in a decreased severity of infection. When compared to thyme and oregano essential oils, thymol and carvacrol exhibit a milder impact on development. This suggests that a portion of sporozoites treated with these compounds managed to survive and develop within cells, whereas sporozoites treated with the essential oils ultimately succumbed.

Natural alternatives operate through a combination of synergistic effects among compounds found in complex mixtures, such as essential oils, or rely on the concentrated nature of single bioactive compounds. In this study, the pure bioactives proved to be less effective than the complex mixture, underscoring the importance of synergistic interaction between natural compounds in enhancing anticoccidial effects. Therefore, formulations combining bioactive compounds might offer a better alternative; however, this will need to be tested in vitro, and if high efficacy is shown, in vivo models will be necessary for efficacy validation.

## 5. Conclusions

In conclusion, this study has shed light on the promising potential of essential oils and their bioactive compounds as anticoccidial candidates. These findings highlight that these natural substances not only inhibit sporozoite invasion but also interfere with the formation of schizonts, a critical phase in the parasite’s lifecycle. Further research on the mechanisms and applications of these natural compounds is necessary, holding promise for safer and more comprehensive anticoccidial solutions in the future.

## Figures and Tables

**Table 1:** Average size of intracellular *E. tenella* after treatment. Data are represented as mean with SEM (*n* = 12). Values are represented as graph in Figure 3.

	Average Size (μm^2^)			
	24 hpi	48 hpi	72 hpi	96 hpi
**Challenged control**	26.70 ± 1.72	56.65 ± 4.70	96.30 ± 9.49	32.8 ± 4.65
**Robenidine 5 ppm**	29.49 ± 1.32	29.90 ± 1.44	18.47 ± 3.38	27.60 ± 1.14
**Salinomycin 1 ppm**	23.21 ± 2.07	17.50 ± 1.13	14.54 ± 1.40	15.66 ± 2.12
**TEO 40 ppm**	25.62 ± 1.40	20.46 ± 1.14	37.01 ± 13.38	18.76 ± 3.81
**OEO 40 ppm**	21.58 ± 1.22	24.30 ± 2.41	30.41 ± 5.89	21.08 ± 3.39
**GAR 40 ppm**	24.54 ± 1.16	26.35 ± 0.60	23.38 ± 1.21	25.67 ± 0.97
**THY 20 ppm**	21.59 ± 0.92	38.26 ± 3.39	45.88 ± 4.71	45.85 ± 7.33
**CAR 20 ppm**	25.45 ± 1.04	45.31 ± 5.16	73.44 ± 9.94	27.71 ± 1.26
**DDS 20 ppm**	24.05 ± 1.60	51.02 ± 3.91	84.870 ± 11.98	27.76 ± 2.23

Thyme essential oil (TEO); oregano essential oil (OEO); garlic oil (GAR); thymol (THY); carvacrol CAR); diallyl-disulfide (DDS).

**Table 2:** Rate of growth of intracellular *E. tenella* after treatment (between 24 and 72 hpi). Data are reported as mean with SEM (*n* = 12).

	Rate (μm^2^/h)
**Challenged control**	1.21 ± 0.08 ^a^
**Robenidine 5 ppm**	−0.23 ± 0.09 ^d^
**Salinomycin 1 ppm**	−0.17± 0.04 ^d^
**TEO 40 ppm**	−0.04 ± 0.03 ^cd^
**OEO 40 ppm**	0.07 ± 0.06 ^cd^
**GAR 40 ppm**	−0.03 ± 0.03 ^cd^
**THY 20 ppm**	0.51 ± 0.10 ^bc^
**CAR 20 ppm**	0.87 ± 0.24 ^ab^
**DDS 20 ppm**	0.97 ± 0.27 ^ab^

^a,b,c,d^ Significant differences among groups (*p* ≤ 0.05). Thyme essential oil (TEO); oregano essential oil (OEO); garlic oil (GAR); thymol (THY); carvacrol CAR); diallyl-disulfide (DDS).

**Table 3:** Level of inhibition of invasion 24 hpi. Data are reported as mean with SEM (*n* = 8).

	Level of Inhibition (%)
**Challenged control**	0.00 ± 9.29 ^bc^
**Robenidine 5 ppm**	51.65 ± 5.44 ^a^
**Salinomycin 1 ppm**	61.40 ± 3.04 ^a^
**TEO 40 ppm**	67.76 ± 5.88 ^a^
**OEO 40 ppm**	55.86 ± 10.63 ^a^
**GAR 40 ppm**	44.70± 10.81 ^a^
**THY 20 ppm**	−26.08 ± 19.74 ^c^
**CAR 20 ppm**	43.05 ± 3.62 ^a^
**DDS 20 ppm**	38.96± 10.47 ^ab^

^a,b,c^ Significant differences among groups (*p* ≤ 0.05). Thyme essential oil (TEO); oregano essential oil (OEO); garlic oil (GAR); thymol (THY); carvacrol CAR); diallyl-disulfide (DDS).

**Table 4:** Quantification of intracellular *E. tenella* DNA by qPCR after treatment. Data are represented as mean with SEM (*n* = 8). Values are represented as a graph in Figure 5.

	Intracellular *E. tenella* DNA (%)			
	24 hpi	48 hpi	72 hpi	96 hpi
**Challenged control**	100.00 ± 9.29	291.99 ± 82.71	676.43 ± 162.14	311.58 ± 27.35
**Robenidine 5 ppm**	48.35 ± 5.44	21.93 ± 4.14	8.06 ± 2.36	12.87 ± 4.75
**Salinomycin 1 ppm**	38.61 ± 3.04	16.28 ± 2.62	13.34 ± 3.97	12.80 ± 3.81
**TEO 40 ppm**	32.24 ± 5.88	13.73 ± 2.88	13.34 ± 2.14	14.25 ± 3.14
**OEO 40 ppm**	44.14 ± 10.63	8.54 ± 2.18	8.98 ± 1.59	9.43 ± 2.77
**GAR 40 ppm**	64.15 ± 12.88	32.12 ± 3.72	16.99 ± 3.78	23.31 ± 7.41
**THY 20 ppm**	126.08 ± 19.74	94.65 ± 30.03	280.62 ± 103.94	250.85 ± 95.30
**CAR 20 ppm**	71.43 ± 14.82	121.33 ± 37.52	166.03 ± 51.33	203.15 ± 71.00
**DDS 20 ppm**	61.04 ± 10.47	208.01 ± 65.58	414.91 ± 33.20	310.68 ± 34.73

Thyme essential oil (TEO); oregano essential oil (OEO); garlic oil (GAR); thymol (THY); carvacrol CAR); diallyl-disulfide (DDS).

**Table 5:** Rate of *E. tenella* DNA replication (between 24 and 72 hpi). Data are reported as mean with SEM (*n* = 8).

	Rate (genomes/h)
**Challenged control**	6.44 ± 0.94 ^ab^
**Robenidine 5 ppm**	−0.84 ± 0.14 ^c^
**Salinomycin 1 ppm**	−0.53 ± 0.09 ^c^
**TEO 40 ppm**	−0.39 ± 0.09 ^c^
**OEO 40 ppm**	−0.73 ± 0.23 ^c^
**GAR 40 ppm**	−0.78 ± 0.30 ^c^
**THY 20 ppm**	3.55 ± 2.45 ^abc^
**CAR 20 ppm**	2.63 ± 1.16 ^ac^
**DDS 20 ppm**	7.37 ± 0.82 ^b^

^a,b,c^ Significant differences among groups (*p* ≤ 0.05). Thyme essential oil (TEO); oregano essential oil (OEO); garlic oil (GAR); thymol (THY); carvacrol CAR); diallyl-disulfide (DDS).

## Data Availability

Data are contained within the article.
